# Queen Charlotte's Hospital and its Nursing Staff

**Published:** 1919-09-27

**Authors:** 


### THE EDITOR'S LETTER-BOX.

**[Correspondence on all subjects is invited, but we cannot in any way be responsible for the opinions expressed by our correspondents, who must give their name and address as a guarantee of good faith, but not necessarily for publication. Correspondents are reminded that brevity of style and conciseness of statement greatly facilitate early insertion.]**

### QUEEN CHARLOTTE'S HOSPITAL AND ITS NURSING STAFF.

WE have received several communications on this matter. One letter is from " A Late Night Sister " at Queen Charlotte's Hospital who left two years ago, and who gives an account of her experience there during three years prior to that date. It has no direct bearing on the present conditions, especially during the last few months and the inner workings of Queen Charlotte's Hospital as dealt with by our correspondent, whose letter we published last week, with the Secretary's statement.

### Two Years Ago ?

*To the Editor of* THE HOSPITAL.

SIR,—With regard to the criticism in this week's HOSPITAL *re* the nursing hours, cleanliness, and defective food supply at Queen Charlotte's Hospital, might I voice my opinion *re* conditions, having within the last five years worked there for a period of well over three years, first as pupil-midwife, then a staff nurse, and finally a sister? I consider some of the statements quite unjust to such a splendid training-school as Queen Charlotte's is. *Re* the dietary. We had for breakfast always, bacon, ham, fish, eggs, or brawn at 6.40 A.M., then at 9.45 in the dining-room there was always plenty of bread and butter or toast, and a glass of new milk. At dinner we had cold or hot meat, and mostly two vegetables, followed by fruit and custard, trifle, or hot pudding. Tea, there was bread and butter. For supper we had meat of some description, followed by a hot pudding with coffee or cocoa. Night nurses had a similar diet. The crockery bears the " Q.C.H." monogram.

*Re* the conditions of bedrooms. Each nurse has a room to herself, and only twice during my three years I was there did I hear of any " Norfolk Howards " being found in these rooms, which were supposed to have been brought in by some nurse who had come in off district work. On both these occasions the matron immediately had the rooms stoved.

During the whole of my time there I never knew a nurse to miss her off-duty time, unless there were two or three nurses off ill. If this was the case the matron always made the time up to the nurse afterwards.

*Re* the amount of cleaning. It is, I believe, what all nurses have to do, wherever they are trained, and I would not have missed the experience of same there for anything. When working in the labour wards materials for cleaning were always supplied to us.

Nurses have come from all parts of the world to train at Queen Charlotte's, and I am perfectly sure they will bear me out in that the work and discipline they learnt at Q.C.H. has never done them any harm. A better committee or matron I never wish to work for.—Yours truly,

LATE NIGHT SISTER.

### The Confirmation of Seven Pupil Nurses,

Next in order came the letter we published below signed with the full names of seven pupil nurses at present engaged at Queen Charlotte's Hospital. We thank our correspondents for the courage they have displayed, for we understand that there are sixty pupil nurses at present engaged at the hospital who have suffered from the conditions complained of, and have each paid £40 for six months' training, portions of which time they have yet to serve, and to face an examination for their certificates of midwifery training. This payment of £40 is reported in some cases, possibly many, to be practically all the available funds of the pupils in question, which may mean life or death to them in the present circumstances. In other words, they are not free agents. Further, necessity makes them philosophical, as the term of endurance to be faced, whatever the discomforts or worse may be, is only a few months, and many of them are under forty.

### The Seven Pupil Nurses' Letter.

SIR,—We beg to express our gratitude for your kind efforts on behalf of the pupils at this hospital. We wish to confirm the very moderate statements made in " Interested's " letter, and, in our opinion, this course is the only one likely to be successful, other methods having failed. The conditions under which the work is carried out in this hospital can only be detrimental to the welfare of the patients.—We are, Sir, yours gratefully,

SEVEN PUPIL NURSES.

### A Round-Robin from Pupil Nurses.

It is understood that a round-robin bearing the signatures of a large majority of the pupils had been prepared, several days before our last issue, for presentation to the House Committee. It was held back (1) because the matron was absent, and (2) because of the statements in THE HOSPITAL of September 20. We congratulate the pupil nurses as a body on this statesmanlike and proper attitude, and hope they will now present their round-robin to the Chairman at the next meeting of the House Committee without fail. They have with them already the sympathy of the nursing profession, the Labour Party, and a host of friends. We think we may assure them that if they make quietly but firmly the protest embodied in their round-robin to the Committee, which many of them have already signed, and all should make it their business to sign, it will be courteously received and every consideration shown to the just demands which they may put forward for redress. Unity is strength, and justice deserves to have behind its enforcement the full authority of all who find themselves wronged. Such a united protest cannot fail to render the maximum of service to the future welfare and status of Queen Charlotte's Hospital, a point to which all the pupils will, we have no doubt, attach deserved importance.

EDITOR THE HOSPITAL.

**The Editor's Letter-Box: Queen Charlotte's Hospital—(*continued*).**

### The Present Situation: by "Interested."

From evidence forthcoming it appears, and cannot be doubted, that the quality and quantity of the daily menu at present have been subject to fluctuation, mainly, we are informed, for the want of systematic supervision. In fact it has been good, bad, or indifferent for the last three months, as luck, or the cook, may ordain. The daily menu, as quoted in detail by your correspondent, still stands as the general basis of dietary, with modifications, that are apparently nobody's business, except those who have to endure, or go without. The hospital monogram on the crockery does not provide for the dearth of handles, or other small but necessary refinements of the table.

The "Bedroom Visitors," in this more recent instance, did not apply to a nurse on district work, but to an unfortunate new pupil. In the interim the "visitors" had increased and multiplied. Stoving is generally recognised to be insufficient, more drastic methods being necessary effectually to exterminate these vermin.

Relating to off-duty, it is a remarkable statement that a nurse "was never known to lose her off-duty." In the best regulated hospitals it is unavoidable; but quite another matter when continually postponed "owing to circumstances," *i.e.* from hospital mismanagement.

Since the publication of the facts now existing, and which are in contradistinction to the pretensions of a modern training-school, an effort has been made "to set the house in order," to institute a certain administrative control, *i.e.* brass polish has been distributed generally, and other important essentials are receiving supervision.

The complaint of the nursing staff is less one of food or brass polish, or the manner in which the authorities conduct a large hospital. The nurses' complaint is against a system which persistently compels a working day of unremitting labour, and of abnormal length. The present working conditions could by good management be equalised and improved, and the pupils provided with ample opportunities for study. The hospital seems to lack enlightened ideas as to what constitutes the happy and successful management of women students in up-to-date training-schools of the best type.

The point of the whole matter appears to me to be, as must be admitted by those who know, that deterioration has insidiously crept into what was once a reasonably good administration. This fact marks an accurate description of the present system and its fruits during the last few months especially. This manifests so serious a descent from efficiency, that it might appear even unjust to some, though it is true in fact.

The Hospital's Original Correspondent.

